# Early Exposure of Bay Scallops (*Argopecten irradians*) to High CO_2_ Causes a Decrease in Larval Shell Growth

**DOI:** 10.1371/journal.pone.0061065

**Published:** 2013-04-15

**Authors:** Meredith M. White, Daniel C. McCorkle, Lauren S. Mullineaux, Anne L. Cohen

**Affiliations:** 1 Massachusetts Institute of Technology/Woods Hole Oceanographic Institution Joint Program in Biological Oceanography, Woods Hole, Massachusetts, United States of America; 2 Department of Biology, Woods Hole Oceanographic Institution, Woods Hole, Massachusetts, United States of America; 3 Department of Geology and Geophysics, Woods Hole Oceanographic Institution, Woods Hole, Massachusetts, United States of America; Aristotle University of Thessaloniki, Greece

## Abstract

Ocean acidification, characterized by elevated pCO_2_ and the associated decreases in seawater pH and calcium carbonate saturation state (Ω), has a variable impact on the growth and survival of marine invertebrates. Larval stages are thought to be particularly vulnerable to environmental stressors, and negative impacts of ocean acidification have been seen on fertilization as well as on embryonic, larval, and juvenile development and growth of bivalve molluscs. We investigated the effects of high CO_2_ exposure (resulting in pH = 7.39, Ω_ar_ = 0.74) on the larvae of the bay scallop *Argopecten irradians* from 12 h to 7 d old, including a switch from high CO_2_ to ambient CO_2_ conditions (pH = 7.93, Ω_ar_ = 2.26) after 3 d, to assess the possibility of persistent effects of early exposure. The survival of larvae in the high CO_2_ treatment was consistently lower than the survival of larvae in ambient conditions, and was already significantly lower at 1 d. Likewise, the shell length of larvae in the high CO_2_ treatment was significantly smaller than larvae in the ambient conditions throughout the experiment and by 7 d, was reduced by 11.5%. This study also demonstrates that the size effects of short-term exposure to high CO_2_ are still detectable after 7 d of larval development; the shells of larvae exposed to high CO_2_ for the first 3 d of development and subsequently exposed to ambient CO_2_ were not significantly different in size at 3 and 7 d than the shells of larvae exposed to high CO_2_ throughout the experiment.

## Introduction

Coastal marine invertebrates are exposed to dissolved carbon dioxide levels that fluctuate on time scales ranging from daily to seasonal as a result of both natural processes and human activities [1]–[3]. These aqueous CO_2_ levels are likely to increase (and pH to drop) in the decades ahead as a consequence of ocean acidification (OA), the uptake of anthropogenic CO_2_ by the ocean [4]–[6]. Since pre-industrial times, increases in anthropogenic emissions of CO_2_ have caused a decrease in surface ocean water pH of 0.1 units, and a decrease of another 0.2–0.3 units is projected by the end of this century [6], [7]. As seawater pH decreases, its calcium carbonate saturation state (Ω) also decreases, and drops in Ω have the potential to make calcification (or shell-building) more difficult, or more energetically costly for the organism [6], [8], [9].

While changes in surface ocean pH are happening on a global scale, the processes affecting coastal and estuarine ocean pH are different and result in more seasonably variable conditions than in the open ocean [2], [10], [11]. Anthropogenic eutrophication is a major factor influencing acidification of coastal and estuarine regions [8], [12], [13]. Eutrophication results in algal blooms, which eventually die, sink to the seafloor, and fuel microbial respiration [14], [15]. This process is exacerbated during summer stratification events [16] and can produce low-pH seasonal bottom waters that are undersaturated with respect to aragonite [11]. Because many coastal bivalve species spawn during summer months, the larvae are exposed to such conditions. Furthermore, because the buffering capacity of seawater is reduced as dissolved inorganic carbon (DIC) increases, it has been suggested that eutrophication could increase the susceptibility of coastal waters to ocean acidification [10]. As atmospheric CO_2_-driven OA and anthropogenic eutrophication increase, the conditions experienced by bivalve larvae will become increasingly unfavorable for shell growth.

There is mounting evidence for negative effects of OA on marine organisms that produce calcareous skeletons, shells, or tests [6], [8], [9], [17], [18]. In particular, marine invertebrate larvae, including bivalves, are vulnerable to a variety of both chemical and physical environmental conditions including decreased pH [19]. For example, mussel and oyster larvae raised in water with pH ∼7.4 were shown to have delayed development to D-stage veligers compared to larvae raised in ambient pH ∼8.1 [20], [21]. Talmage and Gobler [22] showed that hard clams (*Mercenaria mercenaria*) and, to a lesser extent, bay scallops (*Argopecten irradians*) experienced delayed metamorphosis at present-day conditions compared to pre-industrial conditions, suggesting that the 0.1 pH decrease in the last 150 years has already had an impact on bivalve species. OA has been shown to have a negative impact on multiple early life stages of bivalves, including fertilization, D-stage (early) development, later larval development, and juvenile development [8], [23]–[29]. Some species appear to be more tolerant of OA conditions than others. For example, two species of oysters displayed different trends when exposed to a range of pCO_2_ values resulting in Ω_ar_ = 1.3–0.6 for ∼30 days; *Crassostrea virginica* had decreased shell area and decreased calcification with decreasing Ω_ar_, but *Crassostrea ariakensis* showed no change in shell area or calcification [28]. Nonetheless, the majority of work has demonstrated that young bivalves are negatively impacted by the high CO_2_/low pH conditions resulting from ocean acidification. There is evidence that the early larvae of some species are susceptible to OA [20], [21], [30]. Additionally, OA conditions have been shown to negatively affect the survival of bay scallop larvae and the size of competent and post-metamorphic bay scallops [22], [31], [32].

Here, we address the impact of early exposure to elevated CO_2_ on the survival and growth of bay scallop larvae. Bay scallops are ideal as a model organism for this study because of their economic importance as a commercially harvested shellfish and because of their relatively short larval duration (∼2–3 weeks). We also investigate whether transferring the larvae to ambient conditions can reverse the effects of early high CO_2_ exposure. Such a scenario is ecologically relevant in a situation where larvae are spawned in an estuary with relatively high pCO_2_ and are subsequently transported by currents or tides out of the estuary to sites with a lower pCO_2._ We exposed larvae to ambient (nominally 390 ppm CO_2_, pH = 7.93) and high (nominally 2200 ppm CO_2_, pH = 7.39) CO_2_ conditions for a total of 18 days. The high CO_2_ treatment produced a calcium carbonate saturation state that was undersaturated with respect to aragonite (Ω_aragonite_). Such pCO_2_ values and associated saturation states have been observed in summer months in a local estuary (Childs River, Falmouth, MA, USA) where bivalve larvae are found [33]. In addition, we exposed a third group of larvae to high CO_2_ conditions for three days (through the larval D-stage), followed by exposure to ambient CO_2_ conditions for 15 days. We hypothesize that larvae exposed continuously to elevated CO_2_ will grow and develop more slowly throughout the larval period than those exposed to ambient CO_2_ and that survival will also be negatively affected throughout the larval period. We explore whether effects of exposure to elevated CO_2_ conditions during critical initial shell-formation (1–3 days post fertilization) can be altered by a return to ambient CO_2_ conditions.

## Methods

### Adult Collection and Spawning

Adult *A. irradians* (subspecies *irradians*) individuals were collected during spring and summer months from coastal waters around Mashpee, Massachusetts and were held in submerged cages in Little River, an estuarine river near Waquoit Bay, Massachusetts, until needed. The pH at this location ranges from about 8.2 during winter months to about 7.6 during summer months [33]. From monthly water samples, pH (total scale) and pCO_2_ were calculated from measured alkalinity and total DIC. For the summer months when adult scallops were held in cages, monthly pH values ranged from 7.6–7.9. During the same time period, calculated monthly pCO_2_ values ranged from 578–986 ppm. All necessary permits were obtained for the described field studies; a research collection permit was issued by the Commonwealth of Massachusetts Department of Fish and Game, Division of Marine Fisheries. Several days prior to spawning, the adults were brought to Woods Hole Oceanographic Institution, where they were maintained in 16°C flowing seawater and fed daily with Instant Algae Shellfish Diet (Reed Mariculture, Campbell, CA, USA). During feedings, the flowing water was stopped for 2 h and adults were fed at a concentration of 200,000 cells ml^−1^.

Spawning was induced by placing the hermaphroditic adults in a 20°C flowing seawater bath and gradually raising the temperature to a maximum of 25°C, over a period of 3 h. When an individual spawned, it was moved to a beaker of 20°C, 0.35 µm filtered seawater (FSW) and the spawned gametes were examined to distinguish eggs from sperm. To prevent self-fertilization, the water in the beakers was frequently changed. Eggs were rinsed through a 75 µm filter to collect debris, collected on a 20 µm filter, and subsequently pooled. Seawater with sperm was rinsed through a 20 µm filter to collect debris and the sperm was pooled. Eggs were collected from four individuals and sperm was collected from ten individuals. If a scallop released both eggs and sperm, only the eggs were used. Sperm and eggs were each pooled separately into about 1 L of seawater. About 1 ml of sperm was added to the eggs and the embryos were left to develop in the beaker for 11 h, until the larvae were at the swimming gastrula stage [34].

### Larval Culture

When the scallop larvae were 11 h post-fertilization, they were homogeneously suspended in the beaker by gentle plunging with a graduated cylinder [35] and 1 ml was removed to estimate their density. Live larvae were counted at 100X magnification on a gridded slide in which each grid square held 1 µl [36].

Larvae were stocked at an initial density of 30 larvae ml^−1^ and were maintained in 800 ml of 0.35 µm FSW in six 1–l polyethylene cups per treatment, which were previously conditioned in running seawater for at least four weeks. Cultures were fed a daily pulse of laboratory-raised *Isochrysis galbana* (Tahitian strain, T-iso) in the exponential phase of growth at a density of 37,500 cell ml^−1^. This ration has been shown to produce good growth rates and survivorship of bay scallop larvae [36]. Culture water was changed every three days with pre-CO_2_ equilibrated FSW. During water changes, each culture was gently poured through a 20 µm sieve, which caught the larvae. The larvae were rinsed back into the cup and the cup was filled to 800 ml. To maintain a stable temperature, all culture cups were contained in a water bath controlled by an aquarium chiller/heater (T = 22.5±0.3°C).

### Manipulation of Water Chemistry

Water chemistry was manipulated by bubbling cultures with either compressed air or a mixture of compressed air and pure CO_2_. The Ambient CO_2_ treatment was bubbled with compressed air produced by an oil-free, portable air compressor (Porter Cable, Jackson, TN, USA). The High CO_2_ treatment was bubbled with a compressed air/pure CO_2_ mixture precisely controlled using two mass flow controllers (Aalborg, Orangeburg, NY, USA). To create the high CO_2_ treatment, 8.1 ml min^−1^ CO_2_ was mixed with 4.5 l min^−1^ compressed air. Each 800 ml culture cup was bubbled at a rate of approximately 100 ml min^−1^.

Filtered seawater was pre-CO_2_ equilibrated by bubbling with the appropriate air-CO_2_ mixture in 14–l buckets for 24 h prior to filling the 1 l culture cups. Each of the Ambient CO_2_ and High CO_2_ treatment replicate cups was bubbled with ambient CO_2_ compressed air or high CO_2_-compressed air mixture, respectively, for the 18-d duration of the experiment. The High CO_2_ to ambient switch treatment (hereafter referred to as the High CO_2_/Ambient treatment) was bubbled with the high CO_2_-compressed air mixture for the first three days of the experiment, followed by ambient CO_2_ compressed air for the remaining 15 d of the experiment.

### Characterization of Water Chemistry

Prior to water changes, the carbonate chemistry of the pre-equilibrated water in the 14–l buckets was measured. To characterize the carbonate chemistry of the water, pH, total alkalinity, salinity, and temperature were measured. Spectrophotometric pH measurements were made with 2 mM *m*-Cresol purple indicator dye to ensure high accuracy and precision using an Ocean Optics USB4000 Spectrometer with an LS-1 light source and a FIA-Z-SMA-PEEK 100 mm flow cell (Ocean Optics, Dunedin, FL, USA), following the procedure described by Clayton and Byrne [37] and Dickson et al. [38], and using the refit equation of Liu et al. [39]. This method proved to have a precision of ±0.002 pH units.

Samples for total alkalinity analysis were filtered to 0.45 µm, poisoned with saturated mercuric chloride, and stored in sealed glass vials until analysis. Total alkalinities were measured in duplicate via titration with 0.01 N HCl using a Metrohm Titrando 808 and 730 Sample Changer controlled by Tiamo software to perform automated Gran titrations of 1 ml samples. Internal seawater standards were standardized against seawater certified reference materials (supplied by the laboratory of Andrew Dickson, Scripps Institution of Oceanography) and were included in each run.

Salinity was determined using a Guildline model 8400B “Autosal” laboratory salinometer (Guildline Instruments, Smith Falls, Ontario, Canada). The temperature of the water bath was recorded every 10 min by a TidbiT v2 data logger (Onset Computer Corporation, Pocasset, MA, USA) and was also recorded for each replicate culture cup at the time of pH measurements.

Based on the measured values of pH (seawater scale), total alkalinity, temperature, and salinity, we used CO2SYS Software [40] to calculate pCO_2_, Ω_aragonite_, and total DIC using the first and second dissociation constants (K_1_ and K_2_) of carbonic acid in seawater from Mehrbach et al. [41], refit by Dickson and Millero [42].

The High CO_2_ treatment produced carbonate chemistry conditions (Table 1) comparable to those seen in Waquoit Bay during summer months [33] when many bivalve species, including bay scallops, spawn. Bay scallops live in coastal areas and estuaries, which experience seasonal fluctuations in pH and Ω_ar_ influenced by factors other than atmospheric CO_2_. Consequently, the pH and Ω_ar_ currently experienced by coastal bivalve larvae [33] are lower than the values predicted for the open ocean at the end of this century [7], [43].

**Table 1:** Environmental parameters and carbonate chemistry for the Ambient CO_2_ and High CO_2_ treatments during the experiment (mean ± SD).

	Ambient CO_2_	High CO_2_
**Measured parameters**		
Temperature (°C)	22.4±0.3	22.5±0.2
Salinity	32.2±1.1	31.8±0.7
pH	7.93±0.01	7.39±0.03
*A*_T_ (µEq kg^−1^)	2130±68	2114±44
**Calculated parameters**		
*p*CO_2_ (µatm)	509±13	1987±140
[HCO_3_^×^] (µmol kg^−1^)	1779±46	2000±44
[CO_3_^2×^] (µmol kg^−1^)	142±10	46±3
[CO_2_] (µmol kg^−1^)	16±1	61±4
DIC (µmol kg^−1^)	1937±55	2107±47
Ω_aragonite_	2.26±0.14	0.74±0.04

Calculated parameters were calculated from pH and total alkalinity using CO2SYS software [40]. *A*_T_ = total alkalinity; DIC = dissolved inorganic carbon; Ω_aragonite_ = aragonite saturation state.

### Microscopic Imaging and Shell Measurements

At 1, 3, and 7 d, approximately 50 larvae from each culture were preserved in 95% ethanol for subsequent microscopic imaging and shell measurement. At the time of imaging, the preserved larvae were transferred to FSW and viewed at 200X magnification under bright field transmitted light using a Nikon ECLIPSE 50 i POL microscope. Images were captured using a SPOT Insight™ Camera controlled by SPOT Basic Software (Diagnostic Instruments, Inc., Sterling Heights, MI, USA). Using the built-in measurement capabilities of the software program, shell length (the longest dimension parallel to the hinge) was measured for at least 15 larvae from each replicate culture. Mean growth rate (µm d^−1^) for each replicate was calculated as the increase in mean shell length from 1 d to 7 d, divided by the number of days (6 d).

### Survival Estimation

Percent survival was estimated at 1, 3, 7, and 18 d. By 18 d, less than 0.5% of larvae remained alive in any treatment, so both survival and shell size analyses focused on 1, 3, and 7 d. On these days each culture in turn was homogeneously suspended by gently plunging with a graduated cylinder and a known volume (13, 25, and 40 ml for 1, 3, and 7 d, respectively) was removed and concentrated by gently pouring it through a 20 µm sieve. The volumes were chosen to yield a number of larvae (100–200) that could be counted in approximately 30–45 min. All of the live larvae in this volume were counted under a stereomicroscope at 10X magnification. The density of larvae in the removed volume was used to calculate the percent survival based on the initial stocking density of 30 larvae ml^−1^. One replicate culture in the Ambient CO_2_ treatment was discovered to have unreliable survival counts, as a result of a mistake by the counter, so only data from the other five replicate cultures were analyzed.

### Statistical Analysis

All statistical analyses were performed using Systat® 13 Software (Systat Software, Inc., Chicago, IL, USA). Percent survival data were arcsine-square root-transformed prior to statistical analyses. Repeated measures ANOVA tests were run to compare survival among the three treatments at 1, 3, and 7 d and to compare shell length among the three treatments at 1, 3, and 7 d. One-way ANOVAs followed by Tukey's Honestly Significant Difference tests were run to compare survival and shell length among the three treatments at each time-point separately.

## Results

### Shell Length

**Figure 1:**
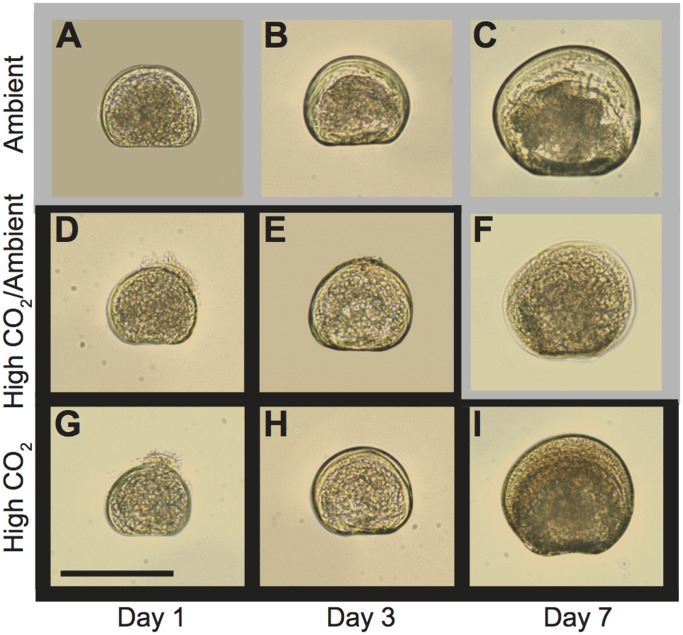
Larval morphology of bay scallops (*Argopecten irradians*) exposed to varied pCO_2_ conditions. Larvae were preserved in 95% ethanol after incubation for 1 d, 3 d, and 7 d in (A-C) ambient CO_2_, (D-F) high CO_2_ for 3 d followed by ambient CO_2_ for 4 d, and (G-I) high CO_2_. Larvae shown represent the mean shell length for each treatment and age. The outline of each image corresponds to the CO_2_ treatment the larvae were experiencing at the time of preservation. Gray = ambient CO_2_, black = high CO_2_. Images are all to the same scale; scale bar = 100 µm.

Larval development (Fig. 1) in all treatments progressed in a sequence typical for this species as described by Belding [34] and Widman et al. [36]. All larvae were fully shelled at 1 d and were post-D-stage by 3 d. While fully shelled, some 1 d larvae had velums that protruded from the shell (Fig. 1d, g); this occurred more frequently in larvae from the High CO_2_/Ambient and High CO_2_ treatments than in larvae from the Ambient CO_2_ treatment.

**Figure 2:**
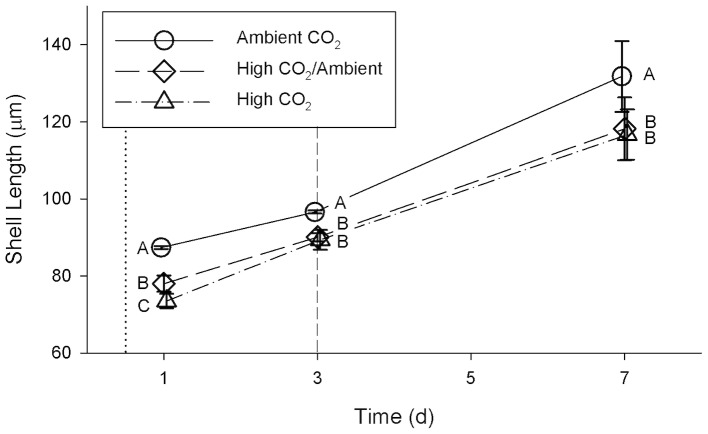
Shell length of larval bay scallops (*Argopecten irradians*) during the first week of larval development. Values are mean ± SD of 6 replicate culture containers. The dotted vertical line indicates the age at the time of inoculation (exposure to CO_2_ treatments); the dashed vertical line indicates the age at which CO_2_ conditions were switched for the High CO_2_/Ambient treatment. Different letters (A, B, C) denote significant differences (*p*<0.05) between treatments at a given age, as determined in one-way ANOVA (Table 2), followed by Tukey’s HSD test.

Exposure to high CO_2_ (Table 1) caused a significant reduction in shell length (Fig. 2) during the first week of development when compared to exposure to ambient CO_2_ (repeated measures ANOVA, Wilk’s Lambda = 0.033; *F = *19.47; df = 6, 26; *p*<0.00001). This pattern was significant for each of days 1, 3, and 7 (One-way ANOVA; Table 2). On day 1, after 12 h of exposure, the mean shell lengths of all three treatments were significantly different from each other (Fig. 2). The difference on day 1 between the High CO_2_/Ambient and High CO_2_ treatments was unexpected, as the larvae were in similar conditions prior to the switch on day 3. On days 3 and 7, the mean shell lengths of larvae from the High CO_2_ and High CO_2_/Ambient treatments were no longer significantly different from each other, despite having been in different conditions since day 3. The mean lengths of shells from the High CO_2_ treatment were 84.1%, 92.5%, and 88.5% of the mean lengths of shells from the Ambient CO_2_ treatment on days 1, 3, and 7, respectively. After day 1, mean (±1 standard deviation) shell growth rates integrated over following 6 days were 7.4±1.5**µm d^−1^ for the Ambient CO_2_ treatment, 6.7±1.2 µm d^−1^ for High CO_2_/Ambient, and 7.2±1.0 µm d^−1^ for High CO_2_; these differences were not significant (one-way ANOVA, *F* = 0.50, df = 2, *p* = 0.62).

**Table 2:** One-way ANOVAs of mean shell length (µm) of *Argopecten irradians* larvae raised in three CO_2_ treatment regimes (Ambient CO_2_, High CO_2_/Ambient, and High CO_2_) at 1, 3, and 7 d; *n* = 6 replicate culture containers per treatment.

Day	Source ofVariation	Type IIISS	df	Mean Squares	*F*-Ratio	*p*-value
Day 1	Treatment	610.774	2	305.387	114.244	<0.001
	Error	40.097	15	2.673		
Day 3	Treatment	187.631	2	93.816	35.058	<0.001
	Error	40.140	15	2.676		
Day 7	Treatment	823.314	2	411.657	6.382	0.01
	Error	967.488	15	64.499		

### Larval Survival

**Figure 3:**
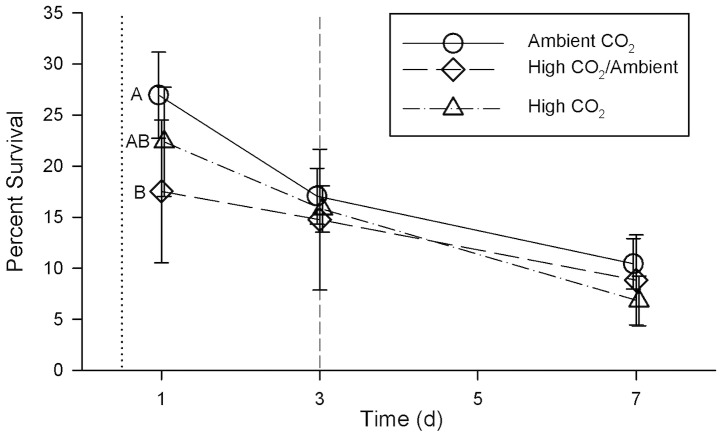
Survival of larval bay scallops (*Argopecten irradians*). Survival is expressed as the percent of larvae surviving from the time of inoculation (age = 0.5 d), during the first week of larval development. Values are mean ± SD of *n* = 5 replicate culture containers for the Ambient CO_2_ treatment and *n* = 6 replicate culture containers for the High CO_2_/Ambient and High CO_2_ treatments. The dotted vertical line indicates the age at the time of inoculation (exposure to CO_2_ treatments); the dashed vertical line indicates the age at which CO_2_ conditions were switched for the High CO_2_/Ambient treatment. Letters denote significant differences (*p*<0.05) between treatments at a given age, as determined in one-way ANOVA (Table 3), followed by Tukey’s HSD test; only the Ambient and High CO_2_ treatments on day 1 are significantly different.

Survival of scallop larvae in the Ambient CO_2_ treatment was consistently higher than survival of scallop larvae in either the High/Ambient or High CO_2_ treatments (Fig. 3) during the first week of development. This overall effect on larval survival was significant in a repeated measures ANOVA (Wilk’s Lambda = 0.38; *F* = 2.50; df = 6, 24; *p = *0.05). However, when the effect was examined for individual days, survival in the Ambient CO_2_ treatment was significantly higher only on day 1 (Table 3, Fig. 3). Survival of larvae in the High/Ambient CO_2_ treatment was not significantly different than in the other treatments at any time during the first week of development.

**Table 3:** One-way ANOVAs of mean percent survival (arcsine-square root-transformed) of *Argopecten irradians* larvae raised in three CO_2_ treatment regimes (Ambient CO_2_, High CO_2_/Ambient, and High CO_2_) at 1, 3, and 7 d; *n* = 5 replicate culture containers for Ambient CO_2_ treatment, *n* = 6 replicate culture containers each for High CO_2_/Ambient and High CO_2_ treatments.

Day	Source ofVariation	Type IIISS	df	Mean Squares	*F*-Ratio	*p*-value
Day 1	Treatment	0.040	2	0.020	3.731	0.050
	Error	0.074	14	0.005		
Day 3	Treatment	0.004	2	0.002	0.502	0.616
	Error	0.056	14	0.004		
Day 7	Treatment	0.012	2	0.006	1.694	0.219
	Error	0.051	14	0.004		

## Discussion

Early exposure (12–24 hours post-fertilization) to high CO_2_ significantly reduced larval shell size (Fig. 2) and survival (Fig. 3) relative to ambient CO_2_ by the time the larvae were 1 d old. The initial reduction in size relative to the Ambient CO_2_ treatment was still evident after the first week of larval development. This suggests that CO_2_ exposure during the first day is critical to shell development. Growth rate from 1–7 d was not significantly affected by CO_2_ exposure, further indicating that growth during the first 24 h post-fertilization determines shell size later in development – the larvae did not increase their growth rate to compensate for initial slow growth. There is evidence that some bivalve larvae (Pacific oysters, *Crassostrea gigas*, and hard clams, *M. mercenaria*) use amorphous calcium carbonate (ACC) to produce the earliest stages of their shell [44], [45]. If this is also true for bay scallops, then it may be a factor in explaining the sensitivity of bay scallop larvae to high CO_2_ conditions during the first day of development, as ACC is more soluble than aragonite and its formation would therefore be less thermodynamically favorable. This demonstration of a significant and lasting CO_2_ effect on shell size within the first day of larval development suggests that other studies on bivalve larval development in which CO_2_ exposure was initiated after the first day of larval development and initial calcification [22], [25], [28]–[31] may have underestimated the magnitude of the effects of high CO_2_ throughout larval development.

Exposure to high CO_2_ caused a significant decrease, relative to ambient conditions, in larval survival at 1 d post fertilization, and a consistent, but not significant, decrease at 3 and 7 d post fertilization. Survival of <20% of individuals on day 7 is low compared to other studies of this species [22], [31], probably due to a combination of not using antibiotics, and calculating survival from initial counts at 12 h post-fertilization rather than 3 d. Mortality during the first 3 d of larval development typically is high and can be variable between culture vessels even when conditions are held constant (Widman, J.C. Jr., personal communication). In our study, survival was highly variable among replicates within treatments and was likely influenced by factors other than carbonate chemistry. This variability may contribute to unexplainable patterns in survival such as the differences on day 1 between High CO_2_ and High CO_2_/Ambient treatments, despite the similar conditions.

A size reduction in scallop larvae exposed to high CO_2_ may have indirect effects on subsequent survival in the field. The age at which a scallop larva is competent to metamorphose is often affected by size [46]. Small scallop larvae may delay metamorphosis, increasing their time in the plankton and risk of mortality from planktonic predators [47]. Previous work has found that exposure of bivalve larvae to high CO_2_ treatments cause delayed metamorphosis, although it is not clear if small size was the cause of the delay [30]–[32]. In addition, if the smaller size of bay scallop larvae exposed to high CO_2_ persisted through metamorphosis into adulthood, the reproductive output of those individuals could be stunted simply because the size of the gonad is proportional to the size of the scallop [48].

The negative effect of high CO_2_ exposure on bay scallop larval size is consistent with negative effects of high CO_2_ exposure on the size of other larval bivalves. The clam *Macoma balthica* has been shown to produce significantly smaller larval shells at 3 d when exposed to seawater with a pH of 7.5 or 7.8 directly after fertilization, compared to a control treatment with pH 8.1 [30]. The same group also reported a similar trend in which *M. balthica* larvae produced (non-significantly) smaller shells from day 5 to 19 when exposed to lowered pH [30]. Similarly, larvae of the blue mussel, *Mytilus edulis*, had shells that, at 2 d, were 12.7% smaller when raised in water with pH 7.6 compared to larvae grown in water with pH 8.1 [25]. Larvae of the oyster *Saccostrea glomerata* showed at 8 d a decrease in shell size of 6.3% at pH 7.8 and 8.7% at pH 7.6 relative to pH 8.1 [29]. By documenting a size effect in the early larval stage of bay scallops, we can better understand the Talmage and Gobler observations of negative effects of high CO_2_ exposure on shell size of 19–20 d old competent scallops [22], [31], [32]. Our results suggest that a decrease in size of competent larvae and set juveniles exposed to high CO_2_ may be a lingering effect of compromised growth in early stages.

Recent work has suggested that marine organisms vary in their potential for evolutionary change in response to high CO2 conditions. For example, when exposed to high CO_2_ conditions (2200 ppm), coccolithophore (*Emiliania huxleyi*) clones that had been raised in high CO_2_ conditions for 500 generations showed a reduced negative calcification response when compared to clones that had been raised in ambient CO_2_ conditions for 500 generations [49]. This indicates that the clones exposed to high CO_2_ conditions for many generations adapted to the unfavorable conditions. Similarly, Sunday et al. [50] showed that larvae of the sea urchin *Strongylocentrotus franciscanus* may have faster evolutionary responses to high CO_2_ than larvae of the mussel *Mytilus trossulus*, despite the fact that *M. trossulus* has a faster generation time and breeder replacement time. Studies of the evolutionary potential of marine invertebrates to adapt to high CO_2_ conditions have been initiated only recently, and bay scallops may be a useful model organism for trans-generational laboratory culturing experiments, as they reach sexual maturity in just one year.

Acidification of coastal waters is affected by atmospheric CO_2_ levels, but it is also strongly impacted by eutrophication [2], [10]–[12], mixing and circulation [11], [51], and input of fresh water [10], [12]. We showed a CO_2_ exposure effect on larval bay scallop growth, but we did not include hypoxia as a treatment. Hypoxia typically co-occurs with high CO_2_ (hypercapnia) as a result of eutrophication in coastal and estuarine waters [2], [12], [52]. Because hypoxia and hypercapnia act synergistically on the responses of marine invertebrates found in such environments [53], it is possible that the interaction of low oxygen and high CO_2_ will affect larval bay scallop development even more strongly than high CO_2_ alone.

The larvae in both High CO_2_ and Ambient CO_2_ treatments maintained similar growth rates from 1–7 d, but the negative effects on shell length of exposure to high CO_2_ during the first 3 d of development were still present after a week of larval development. This result suggests that there is a critical initial window in which CO_2_ exposure is particularly damaging to scallop larvae. Larvae that spawn in coastal and estuarine environments when pCO_2_ is high may have smaller shells, even if they are transported within a few days to waters with lower CO_2_. Aquaculture facilities may need to monitor pCO_2_ in the water used for rearing early larvae to ensure that conditions do not impair growth.
